# Resting and Post-Exercise Ankle-Brachial Index Measurements to Diagnose Asymptomatic Peripheral Arterial Disease in Middle Aged and Elderly Chronic Obstructive Pulmonary Disease Patients: A Pilot Study

**DOI:** 10.14740/jocmr2493w

**Published:** 2016-02-27

**Authors:** Kannayiram Alagiakrishnan, Michael Brokop, Andrew Cave, Brian H. Rowe, Eric Wong, Ambikaipakan Senthilselvan

**Affiliations:** a Division of Geriatric Medicine, Department of Medicine, University of Albert, Edmonton, Canada; b Faculty of Medicine & Dentistry, University of Alberta, Edmonton, Canada; c Department of Family Medicine, University of Alberta, Edmonton, Canada; d Department of Emergency Medicine, University of Alberta, Edmonton, Canada; e Division of Pulmonary Medicine, Department of Medicine, University of Alberta, Edmonton, Canada; f School of Public Health, University of Alberta, Edmonton, Canada

**Keywords:** COPD, Peripheral vascular disease, Ankle-brachial index, Screening

## Abstract

**Background:**

Chronic obstructive pulmonary disease (COPD) patients are at risk for asymptomatic peripheral arterial disease (PAD) because smoking is a risk factor for COPD and PAD. The objectives of this study were to determine the proportion of COPD patients with asymptomatic PAD and to investigate whether the estimated risk of asymptomatic PAD in subjects with COPD differs using resting and exercise ankle-brachial index (ABI) in smokers.

**Methods:**

Using a cross-sectional study design, consecutive smokers > 50 years old were recruited over 2 months from the inpatient units and the outpatient clinics. Subjects previously diagnosed with PAD, unstable angina, recent (< 3 months) myocardial infarction or abdominal, intracranial, eye or lung surgery, and palliative care patients were excluded. Vascular risk factors, ABI (supine and post-3-minute walk supine), self-reported PAD symptoms, and spirometry were obtained. Two measurements of systolic blood pressure on all limbs were obtained using a sphygmomanometer and a Doppler ultrasound, and the ABI was calculated. Data were expressed as means ± standard deviation (SD). Dichotomous outcomes were assessed using Chi-square statistics; P-values of < 0.05 were considered significant.

**Results:**

Thirty patients with no previous diagnosis of PAD were recruited. Mean age was 67.7 years (SD: 10.5). Overall, 21 subjects (70%) had spirometry-proven COPD. Significant ABI for PAD (< 0.9) was seen in 7/21 COPD (33.5%) and 0/9 non-COPD subjects in the supine resting position (P = 0.07), and in 9/21 COPD (42.9%) vs. 0/9 non-COPD subjects after exercise (P = 0.03).

**Conclusions:**

A significant proportion of patients with spirometry-proven COPD screened positive for asymptomatic PAD after exercise. Resting ABI may not be very sensitive to diagnose asymptomatic PAD in COPD subjects. ABI may be a reliable, sensitive and practical screening tool to assess cardiovascular risk in COPD patients. Future large-scale studies are required to confirm this finding.

## Introduction

Chronic obstructive pulmonary disease (COPD) is a common illness in the middle aged and elderly, characterized by frequent exacerbations with declining lung function and increasing mortality with each exacerbation. According to survey studies, COPD prevalence can be seen in up to 18% of adults and it varies due to the difference in diagnostic criteria [1]. By the year 2020, the World Health Organization (WHO) predicts that COPD will become the fifth most common cause of disability and the third leading cause of death worldwide [2].

Cardiovascular disease (CVD) is one of the leading causes of morbidity and mortality in COPD patients. A systematic review suggests COPD has been associated with an increased risk of CVD [3]; however, many patients have undetected early asymptomatic CVD. Risk factors for CVD are still more common than is desirable in COPD subjects. Although peripheral arterial disease (PAD) was previously viewed primarily in terms of being a risk factor for amputation, the significance of its very high risk for cardiovascular morbidity and mortality was generally not well recognized [4]. Objective measurement of cardiovascular risk(s) in asymptomatic patients with COPD would be a helpful clinical tool.

The ankle-brachial index (ABI) has been widely used to detect PAD and may represent a suitable surrogate marker of cardiovascular risk. COPD patients are at risk for asymptomatic PAD because smoking is a risk factor common to both PAD and COPD. Studies have shown PAD is a significant predictor of coronary artery disease (CAD) and carotid artery disease which contributes to the development of myocardial infarction and strokes [5]. A low ABI (indicating disease) is associated with a two-fold higher risk of stroke and myocardial infarction [5]. Studies have shown reduced pulmonary function is independently associated with subclinical atherosclerosis, arterial stiffness, and CAD [6-8]. It is not clear whether having COPD increases the risk of PVD in smokers.

Given these findings, we hypothesized that smokers with COPD would have a higher prevalence of asymptomatic PAD than smokers who do not have COPD. The objectives of this study were to: 1) determine the prevalence of asymptomatic PAD in smoking COPD subjects compared to the smokers without COPD; 2) investigate whether the estimated risk of asymptomatic PAD in subjects with COPD differs using resting and exercise ABI.

## Methods

### Design

This was a prospective cross-sectional pilot study.

### Subjects

Thirty consecutive smokers with or without COPD were enrolled. Subjects were recruited from the outpatient Family Medicine, Senior’s Clinic and Pulmonary/COPD Clinics at Kaye Edmonton Clinic, Garneau Lung Laboratory and the Pulmonary, Geriatric and Family Medicine inpatient units as well as from the University of Alberta Hospital Emergency Department over 2 months. These are all teaching settings affiliated with the Faculty of Medicine & Dentistry at the University of Alberta.

### Inclusion/exclusion criteria

Subjects aged > 50 years of age with a history of smoking were eligible for the study. Subjects who had previously been diagnosed or treated for PAD, having contraindications for spirometry like unstable angina, recent (< 3 months) myocardial infarction, recent (< 3 months) abdominal, intracranial, eye or lung surgery, patients not expected to live 3 months and any patient judged by the attending physician to be at risk of being harmed by completing the study requirements were excluded.

### Enrollment strategy

Patients interested in participating were interviewed by the student research assistant (RA). After obtaining informed consent, clinical historical information including information on vascular risk factors and PAD symptoms was obtained. Additional tests were shown as below.

#### Spirometry

The trained RA performed spirometry to diagnose COPD by using the revised GOLD criteria published in 2011 [9]. Three attempts were recorded and criteria for acceptable completion were employed. A forced expiratory volume in 1 second (FEV_1_) to forced vital capacity (FVC) ratio of less than 0.7 was used to confirm COPD status [10]. All spirometry was reviewed by an expert pulmonary physician.

#### ABI measurement

ABI was performed on all consenting patients. Standardized methodology was used to assess ABI in all subjects. Subjects were rested for 10 min in supine position prior to ABI measurement. Both Doppler ultrasound and sphygmomanometer were used to assess brachial and ankle systolic blood pressure in both arms and legs. Measurement of systolic blood pressure (SBP) on all the four limbs was obtained using a standard sphygmomanometer and a handheld Doppler ultrasound. Two cycles of measurements were obtained for each limb and the mean of the two measurements was taken. ABI was calculated by dividing the higher of the ankle SBP by the higher of the brachial systolic pressure. Supine measurements were repeated after 3 min walking exercise to assess for post-exercise ABI. An ABI of < 0.9 was considered abnormal as per the 2011 American College of Cardiology Foundation/American Heart Association Task Force Practice Guidelines [11, 12].

#### Three-minute walk test (3MWT)

A 3MWT was performed to assess exercise tolerance in each patient. It is a simple test to accommodate patients with respiratory disease and is reflective of activities of daily living [13]. A prospective study showed a good correlation (0.98) between the distances covered in 3 and 6 min in COPD subjects [14]. The functional capacity of the study subjects was performed with a 3MWT and compared between the COPD and no COPD groups.

### Data analysis

Convenience sampling was performed in order to obtain the pilot study subjects. Data were expressed as means ± standard deviations (SDs) or as percent (%), where appropriate. Associations between dichotomous variables were compared using Chi-square statistics or Fisher’s exact test. Statistical analysis was performed using SPSS version 17 (SPSS, Inc; Chicago, IL, USA). A P-value of < 0.05 was considered significant.

### Ethics

Ethics approval was obtained from the University of Alberta Health Research Ethics Board and operations/administrative approval was obtained from Alberta Health Services. Each patient provided informed written consent.

## Results

### Subjects

Thirty smokers were recruited. None had previously been diagnosed with PAD. Mean age was 67.7 years (SD: 10.5), and 11 were females (36.7%). Fifteen subjects (50%) had a history of COPD. Baseline characteristics are shown in Table 1.

**Table 1:** Baseline Characteristics of 30 Smokers Screened in a Canadian Tertiary Care Hospital

Variables	COPD		P-value
	Confirmed (n = 21)	Absent (n = 9)	
Sex (male)	12 (57.2%)	7 (77.8%)	0.42
Sex (female)	9 (42.9%)	2 (22.2%)	0.42
Diabetes	5 (23.8%)	2 (22.2%)	1.00
Hypertension	13 (61.9%)	2 (22.2%)	0.11
Hyperlipidemia	8 (38.1%)	1 (11.1%)	0.44
Cerebrovascular disease	2 (9.5%)	1 (11.1%)	1.00
Alcoholism	2 (9.5%)	0 (0.0%)	1.00
Atrial fibrillation	5 (23.8%)	1 (11.1%)	0.64
Heart failure	2 (9.5%)	0 (0.0%)	1.00
Aortic stenosis	1 (4.8%)	0 (0.0%)	1.00
Chronic renal failure	1 (4.8%)	0 (0.0%)	1.00

COPD: chronic obstructive pulmonary disease.

### Spirometry

Spirometry was performed on all the subjects. Twenty-one subjects were diagnosed to have spirometry-proven COPD and nine subjects had no spirometric evidence of COPD. Mean smoking pack years (COPD 29 ± 16 years vs. non-COPD 32 years ± 19 years) was similar in both groups.

### Screening for PAD

Overall, 7/21 (33.5%) COPD subjects (the study group) and 0/9 (0%) non-COPD subjects (the control group) had an ABI < 0.9 in the supine resting position; 9/21 (42.9%) COPD subjects and 0/9 (0%) non-COPD subjects had an ABI < 0.9 after exercise. Using Fisher’s exact test, there was a trend to an association seen in COPD patients with supine resting position ABI (P = 0.07), and a significant association with exercise ABI (P = 0.03) (Table 2).

**Table 2:** Association Between ABI and COPD in 30 Patients With a Smoking History

ABI < 0.9	COPD (n = 21)	Non-COPD (n = 9)	P-value
Supine (resting)	7 (33.4%)	0 (0%)	0.07
After walk	9 (42.9%)	0 (0%)	0.03

ABI: ankle-brachial index; COPD: chronic obstructive pulmonary disease.

## Discussion

In this study performed on 30 smokers, a significant proportion of patients with spirometry-proven COPD have ABI measures suggestive of asymptomatic PAD. Conversely, patients who smoked yet did not have spirometry-proven COPD have normal ABI measurements and no suggestion of asymptomatic PAD. This finding is important because PAD is a risk factor for vascular diseases of the heart and brain [5]. Few studies have been performed to investigate the relationship between PAD and COPD.

The role of detection of asymptomatic PAD in COPD patients has not been well studied, especially with the resting and post-exercise ABI as a marker of CVD risk. In this study, post-exercise ABI showed a significant association with asymptomatic PAD in COPD subjects, suggesting post-exercise ABI may be more sensitive in diagnosing PAD than resting supine ABI. One study has shown that COPD severity was found to be positively associated with ABI < 0.9; however, that study had not adjusted for vascular risk factors [15]. A study from Taiwan showed a negative association between asymptomatic PAD and lung function [16]. Neither of these studies had control groups or measured the ABI after exercise.

A study by Blum et al, comparing smokers with COPD (n = 23) and healthy volunteers (n = 22, non-smokers) found impaired flow mediated dilatation (P < 0.001); however, no difference in ABI between the two groups was shown [17]. In that study, the controls were non-smokers, whereas in our study, we compared study subjects and controls who were all smokers with and without COPD. Our control group with similar characteristics (smokers) has a less confounding effect due to no difference in baseline characteristics or bias.

Population-based studies showed that a 10% decrease in FEV_1_ is associated with 30% increase in cardiovascular risk of death [18]. Mechanisms like chronic inflammation and endothelial dysfunction may link COPD and CVDs [19, 20]. Since PAD subjects usually present initially with claudication (exercise-induced) symptoms, post-exercise ABI measurement may be better than resting ABI in diagnosing asymptomatic PAD. So, in this study post-exercise ABI has also been done in addition to resting ABI, and in recent studies post-exercise ABI has been shown to provide prognostic information [21, 22].

### Limitations and strengths

This study has limitations. First, since it is a convenient pilot sample, it might not represent the population as a whole. Second, the study was performed at one tertiary care institution in northern Alberta; external validation is required. This study has also some important strengths. First, we recruited control subjects who are smokers without COPD, which was not seen in other studies which investigated this relationship. Second, this is also one of the few studies in which post-exercise ABI measurements after 3MWT was done in addition to resting ABI measurements.

### Conclusions

A significant proportion of asymptomatic PAD, when assessed by post-exercise ABI, is seen in patients with spirometry-proven COPD. The diagnosis of asymptomatic PAD in smokers with COPD is important because it indicates high cardiovascular risk. ABI may be a reliable, sensitive and practical screening tool to assess for cardiovascular risk in COPD patients. Preliminary evidence points out that using the post-exercise ABI may result in greater sensitivity for the identification of asymptomatic or early PAD, but larger future studies are needed to confirm this finding.

### Key points

1) ABI is a risk factor for CVDs. 2) Asymptomatic peripheral vascular disease is seen in COPD subjects. 3) Post-exercise ABI may be a more sensitive test to detect asymptomatic PAD in COPD subjects. 4) Measuring ABI may be a practical screening tool for measuring cardiovascular risk in COPD.
